# Developing a 3-D computational model of neurons in the central amygdala to understand pharmacological targets for pain

**DOI:** 10.3389/fpain.2023.1183553

**Published:** 2023-05-30

**Authors:** Rachael Miller Neilan, Carley Reith, Iniya Anandan, Kayla Kraeuter, Heather N. Allen, Benedict J. Kolber

**Affiliations:** ^1^ Department of Mathematics and Computer Science, Duquesne University, Pittsburgh, PA, United States; ^2^ Department of Neuroscience and Center for Advanced Pain Studies, University of Texas at Dallas, Richardson, TX, United States; ^3^ Department of Engineering, Duquesne University, Pittsburgh, PA, United States; ^4^ Department of Biological Sciences, Duquesne University, Pittsburgh, PA, United States

**Keywords:** neuropathic pain, central nucleus of the amygdala (CeA), agent-based model (ABM), computational model, somatostatin, protein kinase c delta (PKCδ), nociplastic pain

## Abstract

Neuropathic and nociplastic pain are major causes of pain and involve brain areas such as the central nucleus of the amygdala (CeA). Within the CeA, neurons expressing protein kinase c-delta (PKCδ) or somatostatin (SST) have opposing roles in pain-like modulation. In this manuscript, we describe our progress towards developing a 3-D computational model of PKCδ and SST neurons in the CeA and the use of this model to explore the pharmacological targeting of these two neural populations in modulating nociception. Our 3-D model expands upon our existing 2-D computational framework by including a realistic 3-D spatial representation of the CeA and its subnuclei and a network of directed links that preserves morphological properties of PKCδ and SST neurons. The model consists of 13,000 neurons with cell-type specific properties and behaviors estimated from laboratory data. During each model time step, neuron firing rates are updated based on an external stimulus, inhibitory signals are transmitted between neurons via the network, and a measure of nociceptive output from the CeA is calculated as the difference in firing rates of pro-nociceptive PKCδ neurons and anti-nociceptive SST neurons. Model simulations were conducted to explore differences in output for three different spatial distributions of PKCδ and SST neurons. Our results show that the localization of these neuron populations within CeA subnuclei is a key parameter in identifying spatial and cell-type pharmacological targets for pain.

## 1. Introduction

Chronic pain with evidence of central nervous system sensitization is commonly associated with neuropathic pain, which is characterized by “pain caused by a lesion or disease of the somatosensory nervous system” (1, 2), impacts 20% of people who suffer from chronic pain (3, 4), and is difficult to treat (5, 6). Other types of pain, such as nociplastic pain, are also associated with central sensitization, but not any observable neural lesions (7). Although the peripheral nervous system and spinal cord play a critical role in pain chronification and maintenance, the brain contributes heavily to the cognitive and emotional toll in patients.

Within the brain, the amygdala is a key component of the pain matrix (8) and a critical component of pain-depression co-morbidity in humans (9). The CeA has been shown to be a major site of nociceptive processing, receiving input through the “spino-parabrachial nucleus (PBn)-amygdaloid” pathway or through a “thalamic-cortical-basolateral nucleus of the amygdala” relay (10). The central nucleus of the amygdala (CeA) includes the capsular division (CeC), the medial division (CeM), and the lateral division (CeL) subnuclei. Recent studies have highlighted the emerging complexity of CeA cellular heterogeneity along all axes (anterior-to-posterior (A → P), medial-to-lateral (M → L), and dorsal-to-ventral (D → V)) by utilizing a combination of RNA sequencing, projection tracing, and immunoimaging (11–13). This manuscript focuses on two populations of neurons that have been extensively tested in the context of nociception, protein kinase C-delta (PKCδ) and somatostatin (SST). However, this focus should not blind the reader to the emerging complexity of the CeA in the context of nociception. Our understanding of the CeA as a “GABAergic relay nucleus” now includes potentially dozens of unique cell types with different functions based on intra-CeA connectivity and localization along the A → P axis (13–15).

Neurons expressing PKCδ have a pro-nociceptive role in CeA pain output in the mouse cuff-model of neuropathic pain (16). In contrast, SST positive CeA neurons have an anti-nociceptive role. A more recent study supports the antinociceptive function of SST in the chronic constriction injury model (17). However, for SST and PKCδ, others have found contrasting results (18, 19). There is considerable evidence for expression differences in PKCδ and SST as well as other CeA cell-types based on location in the CeC, CeM, and CeL (11–13, 20). Nociceptive inputs to these cells differ as well. Although both receive excitatory projections from the PBn, the type of input (e.g., CGRP positive neurons) is different between SST and PKCδ (10).

We developed the first computational model of PKCδ and SST neurons in the amygdala, based on cell-type physiology data (21). Our model simulates the behaviors and interactions of individual PKCδ and SST neurons within the left and right CeA, each of which is represented by a 2-D grid. The model outputs a measure of neuronal excitability that emerges from these two neuron populations under naïve or injured conditions. Still, the 2-D framework is not capable of capturing spatial heterogeneity in the location and connectivity of PKCδ and SST neurons throughout the CeA.

In this paper, we present our progress towards developing a 3-D computational model. This new model accurately captures the structural features of the CeA and includes spatially heterogeneous distributions of PKCδ and SST neurons estimated from laboratory data along the A → P axis. The 3-D model incorporates a realistic neural network that preserves morphological properties of PKCδ and SST neurons. Our model simulations highlight the importance of these new features and showcase how the 3-D model can be used to investigate spatially targeted pain intervention methods.

## 2. Methods

### 2.1. Model description

Our 3-D agent-based model simulates the behaviors and interactions of 13,000 individual PKCδ and SST neurons within the CeA. The primary emergent model output is the difference in neuronal excitability of two populations of pain-related neurons in the CeA, which varies over time and in response to noxious stimulation. The model was coded in Netlogo3D (V6.2.0) (22) and includes a GUI (Supplementary Figure S1) that allows users to easily modify model parameters, simulate the model, and view output in real time. A detailed model description is provided in Supplementary Appendix A. A summary of model parameters and interpretation of these parameters is provided in Supplementary Table S1.

#### 2.1.1. Spatial domain

**Figure 1:**
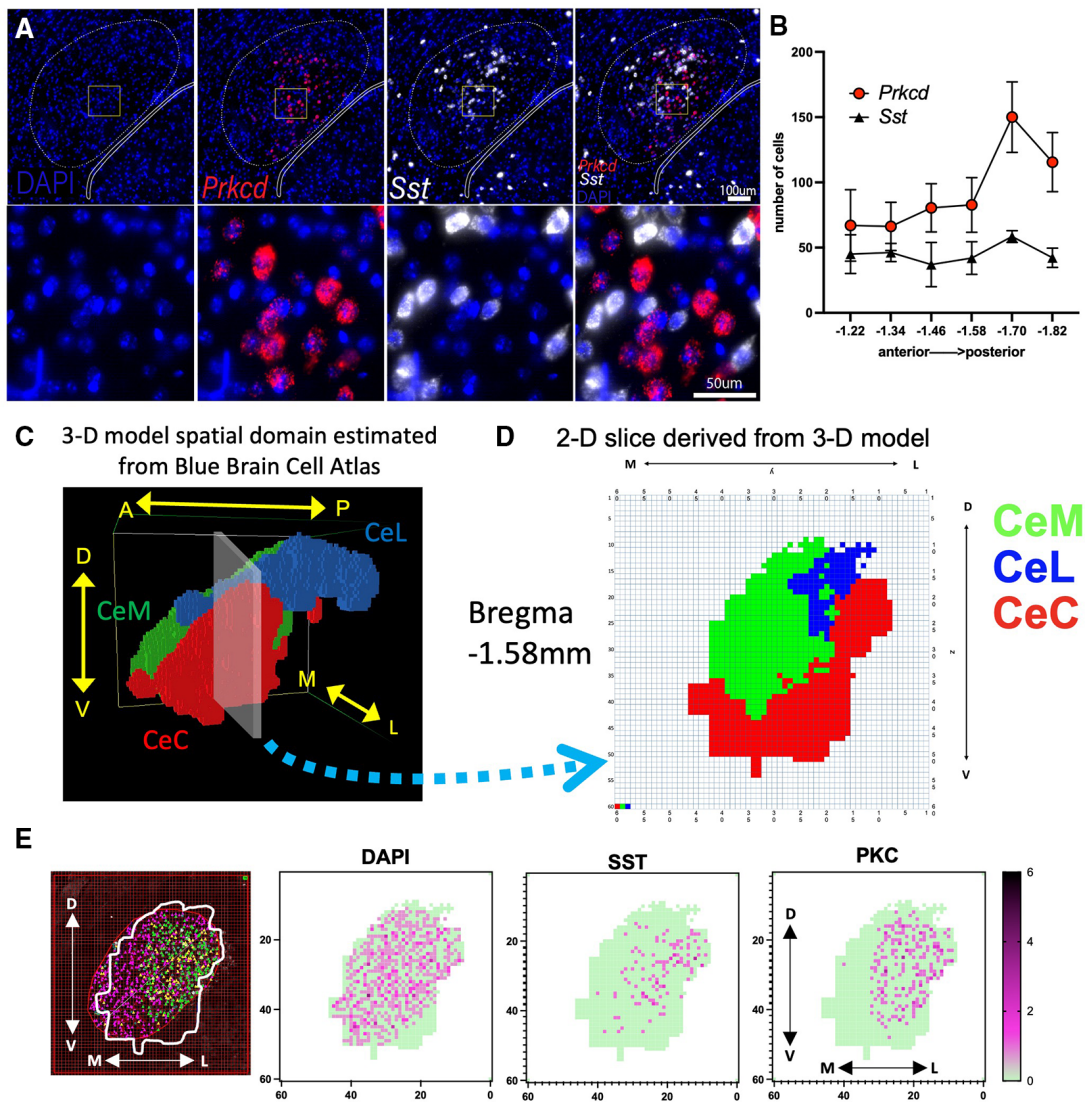
Visualization of PKCδ and SST neurons in the CeA and sub-regions. (**A**) RNAScope *in situ* hybridization was used to quantify the expression of DAPI (nuclei; blue), PKCδ (*Prkcd*; red) and SST (*Sst*; white) in the CeA. (**B**) Graph shows number of cells in the CeA quantified along the rostral (anterior) to caudal (posterior) axis. *n* = 2–5 sections per Bregma location. Axis indicates slice relative to Bregma skull landmark. (**C**) Model patches associated with the right CeA and its subnuclei (CeL, CeC, and CeM) were determined using data from the Blue Brain Cell Atlas. Patches within each subregion are assigned the same color (CeC = red, CeM = green, CeL = blue). Image shows position of model CeA with respect to the anterior to posterior (A → P), medial to lateral (M → L), and dorsal to ventral (D → V) axes. (**D**) 2-D slice representing Bregma location −1.58 mm extracted from 3-D model shown in “C”. (**E**) Illustration of a single −1.58 mm CeA section counted for PKCδ and SST using an overlay of the 2-D representation from “**D**” on a section originally quantified as in “**A,B**”. Shown are cells per 25 × 25µm patch with dark purple representing more cells in a single patch (green = 0 cells in patch). Cells were counted that had DAPI staining (all cells), SST, or PKCδ.

Our model includes a 3-D spatial domain that accurately captures the size and topology of the right CeA and its subnuclei. Focus is placed on the right hemisphere due to the broad consensus that the right CeA is the dominant nucleus in the context of pain (23, 24). The spatial domain was created using publicly available data from the Blue Brain Cell Atlas (25). The atlas provides (*x, y, z*) spatial coordinates for surface vertices of each region in the mouse brain. Coordinates for the CeA were downloaded from the Cell Atlas and imported into NetLogo3D as patches. The *x* axis represents A → P, *y* represents L → M, and *z* represents D → V. Each patch represents 25 µm × 25 µm × 25 µm. Patches associated with the surface and interior regions of each subnucleus were determined and assigned the same color (red = CeC, blue = CeL, green = CeM). The resulting spatial domain yields a realistic representation of the CeA consisting of 16,256 patches (0.25 mm^3^) in the CeC, 7,585 patches (0.12 mm^3^) in the CeL, and 19,970 patches (0.31 mm^3^) in the CeM (Figure 1C). The volume of each subnucleus in the model matches the volume reported by the Cell Atlas within 0.02 mm^3^.

#### 2.1.2. Initialization of model

During the model's initialization, 13,000 agents representing individual PKCδ and SST neurons are created and assigned a cell type (*Type* = PKCδ or SST), location within the CeA, and other variables describing their behavior. The assumption of 13,000 total PKCδ and SST neurons was determined by extrapolating our expression quantities from six cross-sectional slices of the CeA (Supplementary Table S3) to the entire CeA volume. We assumed 60% of the neurons are PKCδ and 40% are SST, which is consistent with findings from our own and others' experiments (20). The location of all neurons is determined at initialization and does not change during a simulation. If the user selects “Uniform Distribution”, each neuron is assigned to a random patch within the CeA, resulting in a spatial distribution that is proportional to the relative volume of each subnucleus (e.g., 37.1% of neurons in the CeC). If the user selects “Non-Uniform Distribution”, each neuron is assigned to a random patch within a specific subnucleus based on wet-lab data. The percent of PKCδ and SST neurons assigned to each subnucleus can be adjusted on the interface.

Each neuron is assigned a firing frequency (*Freq *= Regular Spiking, Late Firing, or Spontaneous) and a damage variable (*d*) tracking the neuron's progress towards sensitization during injury. Each neuron's damage level (*d*) is 0 at initialization, indicating the neuron is unsensitized. Each neuron has a firing rate (*Fr*) in hertz (spikes per second).

After all neurons have been created, a network of uni-directed links is established to allow for the transmission of inhibitory signals between neurons. To create the network, the model randomly selects a neuron and creates a link from this neuron (transmitting neuron) to another randomly selected neuron (receiving neuron) within a distance of $\mathrm{Dis}t_{\max}\;\mu m$. This process continues until all neurons have reached their maximum number of outgoing links $\left(\mathrm{Outgoin}g_{\max}\right)$ or no more suitable links can be made. Parameters describing the maximum length and number of outgoing links assigned to each neuron were determined using published morphology data (20, 26). These data show PKCδ neurons have on average 3.4 dendrites, of which only 30% (∼1 dendrite) connect to other PKCδ or SST neurons. SST neurons have on average 4.8 dendrites, of which 70% (∼3.36 dendrites) connect to other PKCδ or SST neurons. Dendrites originating from PKCδ neurons are on average longer (max length 65 µm) compared to SST (max length 37 µm). In our model simulations, $\mathrm{Outgoin}g_{\max}=1$ and $\mathrm{Dis}t_{\max}=2.6$ patches (65 µm) for PKCδ neurons and $\mathrm{Outgoin}g_{\max}=3$ and $\mathrm{Dis}t_{\max}=1.4$ patches (35 µm) for SST neurons.

#### 2.1.3. Model procedures and output

Model procedures are identical to those in the 2-D model (21). At the start of each time step, the model updates the damage (*d*) and firing rate (*Fr*) of each neuron. Neurons accrue damage when the noxious stimulus is greater than or equal to 120 pA. If a neuron's damage reaches its maximum value (*d* = 100), the neuron is considered “sensitized.” Firing rates of all neurons are updated each time step using the equation

(1) $$Fr_{i}=\frac{100-d_{i}}{100}\cdot X+\frac{d_{i}}{100}\cdot Y$$

where *d_i_* is the neuron's damage level at time step *i* and *X* and *Y* are type-specific random variables describing the firing rates of the neuron in an unsensitized state and a sensitized state, respectively. Distributions for variables *X* and *Y* in Equation (1) were estimated from published physiology data (16, 26).

After the firing rates of all PKCδ and SST neurons are updated, inhibitory signals are transmitted between neurons via the network. The strength of an inhibitory signal is equal to the firing rate of the transmitting neuron. For each neuron, if the total strength of its incoming signal(s) is greater than or equal to 15 Hz (20), the neuron is inhibited during that time step and its firing rate is set to zero $\left(Fr_{i}=0\right)$. If the total strength is less than 15 Hz, the neuron's firing rate does not change.

At the end of each time step, a calculated measure of excitability in CeA pain-related neurons from PKCδ and SST neurons is outputted. The output (*P_i_*) at time *i* is calculated as

(2) $$P_{i}=\sum_{\begin{array}{c} \mathrm{type}=\mathrm{PKC}\delta \\ \mathrm{freq}=\;\mathrm{LF}\;\mathrm{or}\;\mathrm{RS} \end{array}}\frac{d_{i}}{100}\cdot Fr_{i}\;-\sum_{\begin{array}{c} \mathrm{type}=\mathrm{SST}\\ \mathrm{freq}=\;\mathrm{LF}\;\mathrm{or}\;\mathrm{RS} \end{array}}Fr_{i}\;$$

where *d_i_* is a neuron's damage and $Fr_{i}$ is a neuron's firing rate during time step *i*. Equation (2) assumes PKCδ neurons have a pro-nociceptive role in pain-like modulation and SST neurons have an anti-nociceptive role.

All simulations were repeated 100 times due to the stochastic nature of the model. All statistical analyses of model output were conducted in R (27) with P<0.05 considered statistically significant.

### 2.2. Laboratory experiments to determine spatial distributions of PKCδ and SST neurons in CeA

#### 2.2.1. RNAScope *in situ* hybridization

We utilized RNAScope fluorescent *in situ* hybridization to evaluate expression of PKCδ and SST mRNA in the CeA. Five C57Bl/6J mice were perfused with ice cold phosphate buffered saline followed by 4% paraformaldehyde. Brains were extracted and postfixed for 3 h at 4°C. Brains were transferred to 30% sucrose and stored at 4°C for 5 days before being flash frozen and sectioned on a cryostat. 20*μ*m coronal sections were collected and stored in antifreeze at −20°C. Representative sections from across the rostral-caudal axis of the CeA (Bregma −1.22 mm to −1.82 mm) were mounted on Fisher SuperFrost Plus slides (#12-550-15). Twenty-four hours after mounting tissue, slides were immersed in two 5-minute xylene baths followed by two 2-minute 100% ethanol baths. Tissue was then treated with protease III (ACDBio Inc.) in HybEZ oven for 20 min at 40°C. RNAScope *in situ* hybridization was performed according to manufacturer's instructions (ACDBio Inc.). Probes for *Prkcd* (PKCδ) and *Sst* (SST) were hybridized to tissue in HybEZ oven at 40°C for 2 h. Slides then underwent a series of signal amplification (AMP 1, AMP 2, and AMP 3) and attachment of a TSA-based fluorophore (Perkin Elmer, #NEL744B001KT and #NEL745B001KT), all in HybEZ oven at 40°C, with wash buffer baths between each step. Slides were airdried and cover slipped using VectorLabs anti-fade DAPI mounting medium (#H-1500-10).

#### 2.2.2. Image analysis

Images were captured on a Nikon Eclipse Ti2 microscope within 48 h of *in situ* hybridization (Figure 1A). Images with good structural integrity (i.e., no holes in section over the CeA) were analyzed using NIS-Elements Research software. A region of interest (ROI) was hand drawn around the CeA based on rostral-caudal position of the sections referenced from the Allen Brain Atlas (28, 29). *Prkcd* (PKCδ) and *Sst* (SST) were then quantified across the collected sections using two different methods.

First, the CeA was initially quantified using the Miyawzawa method (30). After using an oval to outline the CeA, positive cells were identified as a DAPI labeled nucleus surrounded by at least three puncta of *Prkcd* or *Sst* marker. Positive cells within each subnuclei ROI were counted by hand. Relative abundance of positive cells was normalized to the number of DAPI labeled cells (Figure 1B). A limited amount of this data set specifically for the CeC counted with the Miyazawa method was published recently showing *Prkcd* and *Sst* along the anterior-to-posterior axis (31). Next, we recounted these sections above using a novel second method.

We utilized 2D boundaries of the CeC, CeL, and CeM derived from the Blue Brain Cell Atlas and our 3-D model to create a ROI for each anterior-posterior slice and estimate the expression of *Prkcd* and *Sst* within each subnucleus (Figure 1D). We identified the corresponding position of each slice along the A → P axis of the model's spatial domain (Figure 1C) and exported the 60 × 60 grid (model “slice”) with colored patches indicating the location of the CeC, CeL, and CeM at this location. Each model slice was centered over the previously counted CeA images (Figure 1E). The PKCδ and SST that were previously marked using the method above were counted only within the ROI established by a 2-D Blue Brain Cell Atlas overlay. Cell counts were completed independently by two different researchers.

## 3. Results

### 3.1. *Prkcd* and *Sst* expression in CeA subnuclei

We utilized fluorescence *in situ* hybridization to approximate PKCδ and SST mRNA expression across 6 A → P locations of the CeA (Figure 1A). The CeA in each section was originally identified and outlined using the Miyawaza method (30) (Figure 1B). The Miyawaza method provides the opportunity to divide the CeA subnuclei systematically, but it does not fully match the estimated shapes and sizes of the subnuclei as seen in the model's spatial domain estimated from the Blue Brain Atlas (Figure 1C). To estimate the expression of *Prkcd* and *Sst* more accurately within the CeC, CeM, and CeL, we derived 2D slices from the spatial domain (Figure 1D). The outline of each CeA was then superimposed on the sections previously counted and a 60 × 60 grid was used to count cells. An example of this process for a single section is shown in Figure 1E. All sections were recounted using this method with each positive cell mapped using coordinates corresponding to the model's spatial domain (Supplementary Figure S2); average numbers were calculated for each cross-sectional slice (Supplementary Table S3).

### 3.2. Impact of spatial heterogeneity in PKCδ and SST neurons on model output

The model was simulated 100 times each using a uniform or two different non-uniform spatial distributions of PKCδ and SST (Table 1, Supplementary Figure S3). In the uniform distribution, PKCδ and SST neurons were distributed within the subnuclei based on the relative volume of each subnucleus. In “Non-Uniform A”, the percentage of PKCδ and SST cells in each subnucleus was estimated from our own lab experiments (Section 3.1). In “Non-Uniform B” we used previously published data (13) to determine the percentage of PKCδ and SST cells in each subnucleus. All three distributions used the same total number of neurons (13,000 neurons) and same percentage of neurons assigned to each cell-type (60% PKCδ, 40% SST). The only difference between the distributions was the localization of these neurons within the subnuclei of the CeA (Supplementary Figure S3).

**Table 1:** Spatial distributions of PKCδ and SST neurons used in model simulations. Table shows the percentage of PKCδ and SST neurons assigned to the CeC, CeL, and CeM within the CeA. The uniform distribution results when neurons are randomly assigned a location within the CeA given the relative volume of the three sub-regions estimated from the Blue Brain Atlas. In the non-uniform distributions, a specified percentage of PKCδ and SST neurons are assigned to each subnuclei. Percentages in the non-uniform distributions were estimated from our lab experiments (Section 3.1) and previously published data (13).

Spatial distribution of neurons	PKCδ (%)			SST (%)			Reference
	CeC	CeL	CeM	CeC	CeL	CeM	
Uniform	37.1	17.3	45.6	37.1	17.3	45.6	NA
Non-Uniform A	41.1	17.5	41.4	41.6	12.8	45.6	Estimated from our lab experiments (Section 3.1)
Non-Uniform B	49.8	44.5	5.7	6.7	51.3	42.0	Estimated from published data (13)

**Figure 2:**
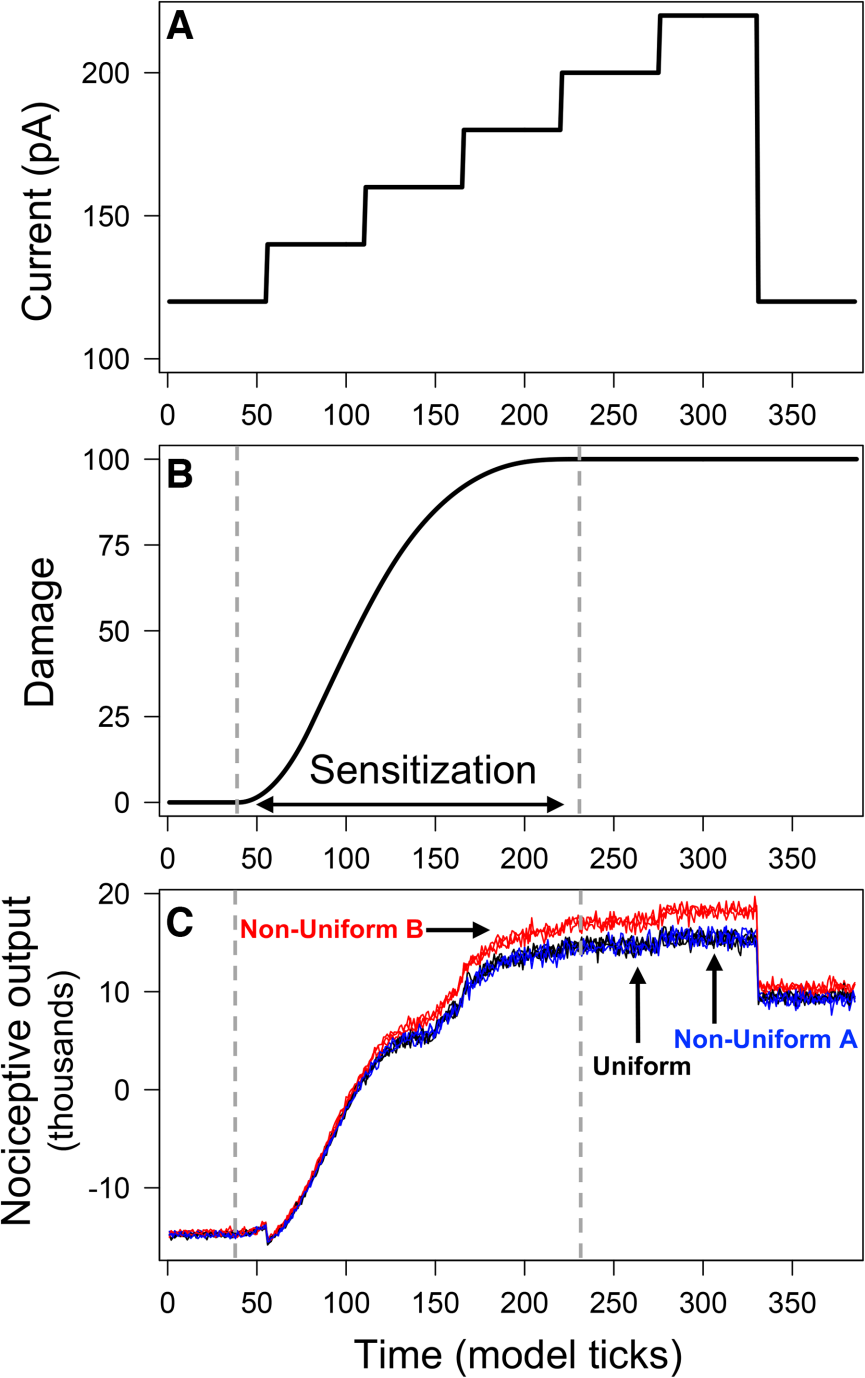
Output from model simulations with uniform and non-uniform distributions of PKCδ and SST neurons. Model was simulated 100 times each using a uniform distribution of neurons and two different non-uniform distributions. All simulations used the ramping current in (**A**) as the stimulation. Average neural damage in (**B**) increases between times t=40 and t=230, indicating neural sensitization. Nociceptive output from PKCδ and SST neurons for n=3 model simulations is plotted in (**C**) for each of the distributions.

Model simulations used a “ramping current” that starts at 120 pA (defined as a “baseline” noxious stimulation) and increases in increments of 20 pA until it reaches 220 pA, after which it returns to 120 pA (Figure 2A). During times t=40 to t=230, the average damage across all neurons increases, indicating the sensitization of neurons due to injury (Figure 2B). At t=230, all neurons have accrued maximum damage and are considered fully sensitized. Figure 2C displays predictions of nociceptive output from PKCδ and SST neurons for n=3 simulations of each spatial distribution. Before sensitization (t<40), nociceptive output is negative [range: (−15,700, −13,600)] and interpreted as the absence of active pain-related output. Increases in nociceptive output are interpreted as increases in pain-related output from the CeA. In all scenarios, nociceptive output increases in response to increases in stimulation (representative of evoked cellular responses). Nociceptive output decreases but remains elevated when current returns to 120 pA (representative of spontaneous nociception after central sensitization). While model predictions are nearly identical for Uniform and Non-Uniform A, model predictions using Non-Uniform B are significantly greater during and after sensitization (n=10,P<0.05).

Differences in output across the three distributions are attributed to differences in emergent network properties (Supplementary Table S2). As expected, the resulting network properties for Uniform and Non-Uniform A are similar. Non-Uniform B resulted in fewer total links (i.e., connections between neurons) on average compared to Uniform and Non-Uniform A. The average number of incoming links to SST neurons was higher for Non-Uniform B compared to the other two distributions. On the other hand, the average number of incoming links to PKCδ neurons was lower for Non-Uniform B. Thus, with Non-Uniform B, we saw increased inhibition of SST neurons and decreased inhibition of PKCδ neurons via the network, resulting in greater nociceptive output compared to the other two distributions (Figure 2C).

### 3.3. Impact of spatially targeted inhibition and activation on pain

One of the advantages of the 3-D model is the ability to target cells in different locations within the CeA to investigate the potential for spatially separate populations to differently impact nociception. We performed spatially targeted inhibition and activation of PKCδ and SST neurons *in-silico* and measured the impact of these cell-type manipulations on nociceptive output during sensitization. The anterior CeA was defined as all CeA patches with x≤26. The posterior CeA was defined as all CeA patches with x≥48. All simulations used the “ramping current” in Figure 2A. At time t=145, we initiated inhibition (or activation) of select neurons in either the posterior or anterior regions of the CeA by modifying the neurons' firing rates. When a neuron is inhibited, its firing rate is 0 Hz. When a neuron is activated, its firing rate is 15 Hz, which is the minimum signal strength needed to inhibit a connecting neuron (20). All changes in neural firing rates due to targeted inhibition (activation) remained in effect for 10 timesteps. We performed 100 replicate simulations of all inhibition and activation scenarios.

**Figure 3:**
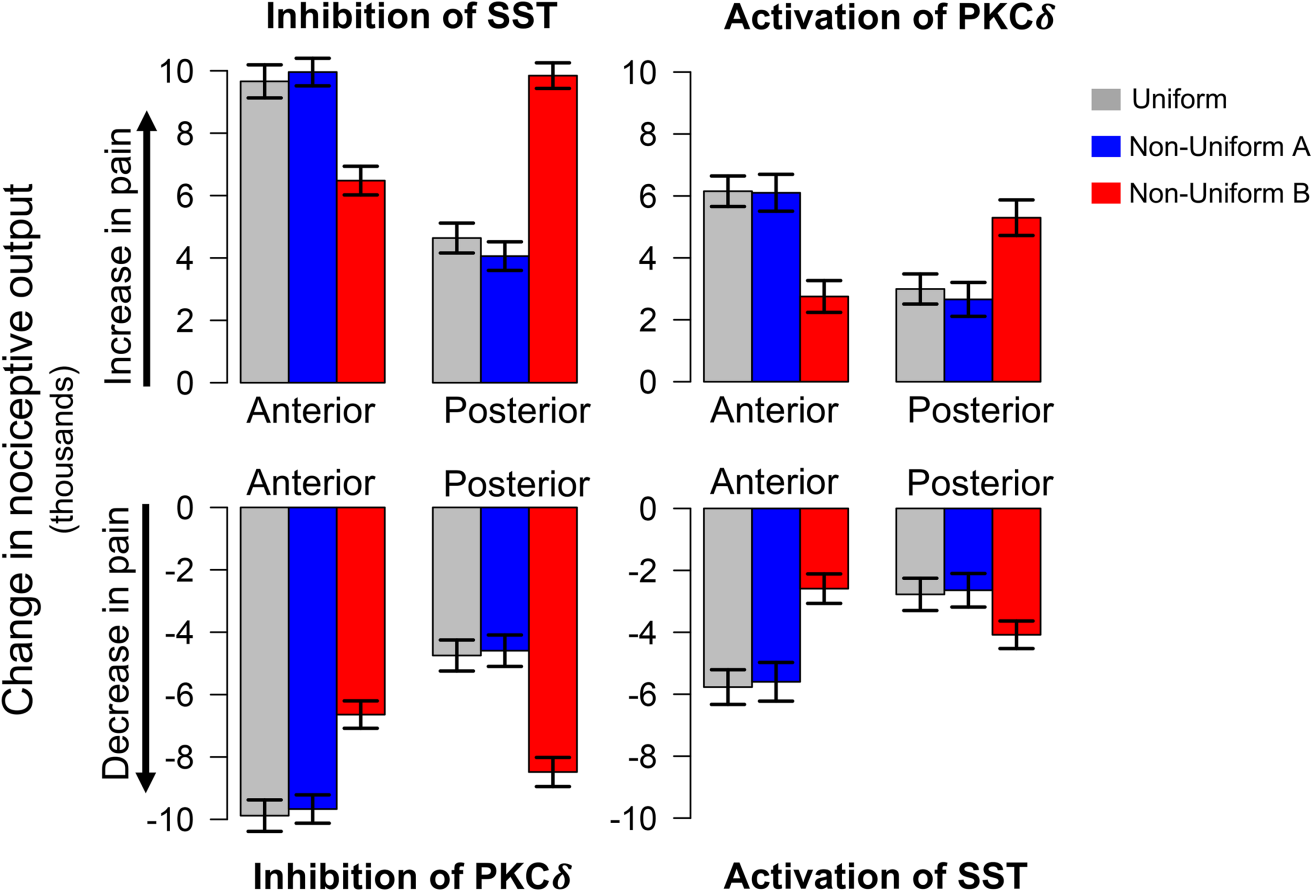
Model predicted changes in pain due to spatially targeted cell inhibition and activation. Bars indicate the change in nociceptive output during sensitization (t=150) when select neurons in either the anterior or posterior regions of the CeA are inhibited or activated. Inhibition of SST and activation of PKCδ result in an increase in pain (top row), while inhibition of PKCδ and activation of SST result in a decrease in pain (bottom row). All scenarios were simulated 100 times each for the uniform and two non-uniform distributions of neurons. Error bars indicate ±1 standard deviation. Across all distributions, model predications show significantly different changes in pain for cellular manipulation of anterior vs. posterior regions. Additionally, in all eight scenarios above, model predictions with Non-Uniform B yielded significantly different changes in pain compared to Uniform and Non-Uniform A (P<0.001).

Figure 3 displays the change in nociceptive output attributed to activation or inhibition of select neurons compared to model predictions without cellular manipulation (Figure 2C). As expected, inhibition of SST and activation of PKCδ increase model output while inhibition of PKCδ and activation of SST decrease model output. For each spatial distribution, we see significantly different changes in model output when comparing corresponding results for anterior vs. posterior (*P* < 0.001), suggesting that the location of PKCδ and SST cells along the A → P axis influences their respective contributions to nociception.

In each of the eight scenarios (e.g., inhibition of SST in Anterior in Figure 3), results from Uniform (grey bars) and Non-Uniform A (blue bars) are similar. However, across all scenarios, Non-Uniform B (red bars) yields significantly different results compared to the other two distributions (*P* < 0.001). This difference suggests that the spatial distribution of neurons is an important parameter in furthering our understanding of spatially dependent pharmacological targets. For example, if the goal is to decrease pain through inhibition of PKCδ, simulations with Non-Uniform B suggest targeting the posterior CeA for optimal results. On the other hand, simulations with Uniform and Non-Uniform A indicate optimal results are obtained through targeting the anterior CeA. Similar differences exist for activation of SST, inhibition of SST, and activation of PKCδ (Figure 3).

## 4. Discussion

Although 3-D models of the basolateral amygdala (BLA) in the context of fear conditioning have been published (32), this manuscript shows the first example of a 3-D CeA model relevant to the study and understanding of cellular nociception. The goal of this process is to build a pipeline for increasingly complex models that accurately recapitulate the complexity of the CeA.

In our simulations, we used three different distributions of PKCδ and SST neurons in the CeA estimated across the A → P axis. The uniform distribution was selected as a comparison to the non-uniform distributions that are expected in the brain. In the uniform distribution, PKCδ and SST neurons were uniformly distributed within the CeA, resulting in distributions proportional to the relative volume of each subnucleus as quantified by the Blue Brain Cell Atlas. Next, we used our own laboratory data and one published data set to create two non-uniform distributions. Our estimates (Non-Uniform A) closely matched the uniform distribution. In contrast, data from McCullough (Non-Uniform B) differed significantly from our own estimates of PKCδ and SST expression within the CeA subnuclei (13). There were two notable differences. First, our non-uniform distribution showed similar PKCδ percentages in the CeM and CeC while McCullough showed relatively little in the CeM. This difference is driven by two sections in our data set that are posterior in the brain (Supplementary Figures S2E,F) with very little CeC and high quantities of PKCδ. These counts skew the overall distribution of PKCδ to ∼50:50 split between CeC and CeM. Second, the overall percentage of cells in the CeL is significantly higher in the McCullough distribution, a phenomenon that may be due to inclusion of sections from Bregma −0.8 to 1.8 mm in this study. A related reason may be differences in how CeAs were defined. We counted cells based on a pre-defined CeA region defined using another method (30), whereas McCullough directly overlaid the Allen Brain Atlas sub-region map on the tissue section. Either way, these differences highlight a challenge in the common practice where only a few sections are used represent a structure like the CeA that varies considerably in size, shape, and cell number along the A → P axis.

Our results suggest that manipulation of the anterior vs. posterior CeA causes a disproportional impact on nociceptive output from two populations of CeA neurons. This illustrates the value of computational models in exploring hypotheses and the potential impact of A → P differences in PKCδ and SST cells on pain-related output from the CeA. A → P differences in amygdala cell-type expression, projections, and function have been documented (13–15, 33–35). Appetitive behaviors are differentially modified based on BLA → CeA projections along the A → P axis in a cell-type specific manner (14). Individual cell types, such as *Calcrl*-expressing cells, show different behavioral reactions (locomotion and flight vs. passive coping) depending on whether the cells are activated in the anterior vs. the posterior CeA (36).

Data suggest PKCδ in the CeA is either pronociceptive (16) or antinociceptive (18, 19) in animal models. The PKCδ neurons that reduce pain-like behavior tend to be more posterior in the CeA (18). In inflammatory pain models in mice, the topographical organization of CeA neurons depends on their efferent projections (37). PKCδ excitability changes on the A → P axis with regular spiking neurons having a higher firing rate in the posterior CeA (26). Taken together, these data suggest that there is a population of hypo-excited PKCδ neurons in the anterior CeA that drive the pronociceptive effects when all PKCδ neurons in the CeA are activated (16). These discrepancies are challenging to resolve in laboratory experiments alone. The value of computational modeling is the ability to address these questions quickly prior to investment in resource-heavy wet-lab experiments.

The 3-D model of the CeA presented here is part of an on-going project to synthesize physiology data for dozens of CeA cell-types to create a “complete” model of the CeA in the context of central sensitization (or other CeA-driven phenomena). As illustrated here, limited sampling of different CeA cell populations along the A → P axis leads to disparate results. This limited sampling has been driven in the field by the limited number of Cre-driver lines and availability of specific antibodies. Studies beyond the scope of this manuscript are needed to sample the CeA more thoroughly in all A → P sections rather than assuming homogeneous distributions of cells across the structure. Furthermore, a number of exciting papers (11, 12) have illustrated the potential for distinct sub-sub-types of cells in the CeA. In other words, even assuming that all SST neurons are the same is an oversimplification (12) as is the assumption that nociceptive output from the CeA can easily be calculated from a simple summation of SST and PKCδ firing rates. Using sequencing, physiology, and functional behavioral studies, we anticipate that the next iteration of our 3-D CeA model will better represent the complexity of this structure in the context of nociception and neuropathic pain.

## Data Availability

The original contributions presented in the study are publicly available. This data can be found here: Open Science Framework (OSF) public repository (https://osf.io/xnrqa/, doi: 10.17605/OSF.IO/XNRQA).
