# Supplementary material for: Developing a 3-D computational model of neurons in the central amygdala to understand pharmacological targets for pain
Source: Front Pain Res (Lausanne). 2023 May 30;4:1183553. doi: 10.3389/fpain.2023.1183553 (PMC10270735; doi:10.3389/fpain.2023.1183553)
Supplement: Supplementary file 1 [file Datasheet1.pdf]

Appendix A: Detailed Model Description

Here we present a detailed description of our 3-D agent-based model (ABM) of pain-related neurons the central nucleus of the amygdala (CeA). This section is written in accordance with the Overview, Design concepts, and Details (ODD) protocol, which is a standard format for describing ABMs. This 3-D ABM is an extension of our existing 2-D ABM (Miller Neilan et al.

1. Purpose

The purpose of this ABM is to simulate realistic spatial distributions, behaviors, and interactions of two types of pain-related neurons in the CeA. We use the model to study the impact of spatial heterogeneity in these neuron populations on emergent system-level output. The primary system-level model feature is a measure of nociceptive output from the CeA. The ABM includes a graphical interface (**Fig. S1**) that allows researchers to initialize the model with different distributions of neurons and view emergent model output in real time during a simulation.

2. Entities, state variables, and scales

The model consists of 13,000 agents representing pain-related neurons within the right CeA. Each agent represents an individual neuron that expresses either protein kinase C δ (PKCδ) or somatostatin (SST) and is responsive to neuropathic injury in animal models. The 3-D spatial domain consists of 43,811 patches within a 75x60x60 grid and accurately captures the structural features of the CeA and its subnuclei (**Fig. 1C**). Patches associated with each CeA subnuclei were determined using publicly available data from the Blue Brain Cell Atlas (Ero et al. 2018). All patches within the same sub-nucleus are assigned the same color (CeC = red, CeM = green, CeL = blue). Each patch represents $25\mu m$ x $25\mu m$ x $25\mu m$. Directed links between agents represent unidirectional, inhibitory connections between neurons. Each model tick represents one time step.

Each of the 13,000 neurons has ten variables (**Table A1**) defining its properties and behaviors. During initialization, each neuron is assigned an (x, y, z) location within the CeA spatial domain and a protein-expression type (*Type*) equal to either PKCδ or SST. A neuron's location and protein-expression type remain constant throughout a simulation. Each neuron has a firing frequency pattern (*Freq*) equal to regular spiking (RS), late firing (LF), or spontaneous (Spont). Each neuron has three variables (d, t_L, t_S) related to "damage". The damage variable (*d*) represents the percentage of total damage accumulated by a neuron in response to external stimulation. The rate at which a neuron accumulates damage during stimulation depends on its damage latency period (t_L) and sensitivity (t_S). Additionally, each neuron has three variables related to its connectivity with other neurons within the CeA. Two of these variables are the neuron's number of incoming connections (*Num-In-Link*) and number outgoing connections (*Num-Out-Link*). The third variable is the neuron's inhibition status (*Inhibited*), which is a binary variable indicating whether the neuron is inhibited or not. Lastly, each neuron has a firing rate (*Fr*) describing the frequency in hertz (spikes per second) of the neuron's action potentials.

Each connection (i.e., directed link) in the neural network has three variables (**Table A2**) describing how inhibitory signals are transmitted between its endpoints. Each connection has a transmitting endpoint ($end1$) equal to the ID of the agent that is the source of the signal and a receiving endpoint ($end2$) equal to the ID of the agent that is the destination of the signal. Each connection has a signal strength (*str*) equal to the firing rate of the neuron associated with its transmitting endpoint. When used, the neural network is established during the model's initialization and does not change during a simulation. A user can choose not to use the neural network by switching the 'Network' toggle on the user interface to "Off".

3. Global Variables and Input Data

Global variables $Outgoing_{max}$ and $Dist_{max}$ control the size of the neural network. $Outgoing_{max}$ specifies the maximum number of outgoing connections assigned to each neuron. $Dist_{max}$ specifies the search radius used by a neuron when forming links with other neurons. Both $Outgoing_{max}$ and $Dist_{max}$ are non-negative values that depend on the neuron's type (PKCδ or SST) and can be specified on the model's interface.

The timing, duration, and magnitude of stimulation (measured in pA) must be input as a file consisting of integer values, ranging from 0 to 220. During initialization, the values in the file are read one at a time and stored as a global vector, S, where the S_i represents the stimulation (pA) applied during the ith time step. In our simulations, the current ranges from 120 pA to 220 pA.

Lastly, the global variable S_{tot} tracks the cumulative number of time steps during which stimulation is greater than or equal to 120 pA. During initialization, this variable is set to 0, and is increased by 1 during times steps in which $S_i \geq 120$.

4. Process overview and scheduling

The following processes occur in the order listed during each simulation of the ABM.

1. Input data is provided by user.
2. Initialization of model:

    i. Read in patch coordinates associated with CeC, CeL, and CeM from a file and create spatial domain representing the CeA.

    ii. Create 13,000 agents representing neurons in the CeA.

    iii. Assign attributes to all neurons.

    iv. Create network of directed links between neurons (if network is turned on).

    v. Create vector specifying stimulation history.

3. During each time step:

i. Update the cumulative stimulation variable, S_{tot}.

ii. Update the damage level of each PKCδ and SST neuron.

iii. As needed, SST spontaneous neurons with maximum damage ($d = 100$) are converted to regular spiking.

iv. Update the firing rate of each PKCδ and SST neuron using probability distributions estimated from laboratory data. Distributions are dependent on neuron type, firing frequency, damage level, and the stimulation value.

v. Use network to send inhibitory signals between neurons. If a neuron is inhibited, its firing rate is set to zero.

vi. Update all system-level observations.

5. Design concepts

5.1 Emergence

The primary emergent feature of the model is a measure of nociceptive output from the CeA that evolves over time and in response to noxious stimulation. In the model, nociceptive output is measured as the difference between the cumulative firing rates of pro-nociceptive PKCδ neurons and the cumulative firing rates of anti-nociceptive SST neurons in the CeA. During each model time step, individual neuron properties are updated and then a system-level measure of nociceptive output is calculated.

Other emergent features of the model include the size of the neural network (i.e. total number of links), average number of incoming links for PKCδ and SST neurons, and average number of outgoing links for PKCδ and SST neurons.

5.2 Adaptation

A damage accumulation model is used in the ABM to allow neurons to adapt to a sensitized state over time and during stimulations measuring 120 pA or higher. Neurons start with zero damage and accrue damage at individual rates and only during time steps in which $S_i \geq 120$. As a neuron's damage increases, the neuron transitions towards a sensitized state and its firing rate is adjusted accordingly. A neuron is considered fully sensitized when its damage variable has reached maximum value ($d = 100$).

Based on lab data, the firing frequency of some SST neurons changes with injury. Experimental results show the quantity of RS SST neurons increases from 27% pre-injury to 48% post-injury, while the quantity of spontaneous SST neurons decreases from 55% pre-injury to 34% post-injury (Hunt et al. 2017). We assumed this change in distribution is through conversion of spontaneous neurons to RS neurons. Thus, in the ABM, individual spontaneous SST neurons that have accumulated maximum damage ($d = 100$) are converted to RS one at a time until the quantity of regular spiking SST neurons has reached 48%.

5.3 Interaction

Interaction occurs in the model when a neuron is inhibited by one or more other neurons. If the sum of the inhibitory signals transmitted to a neuron during a single time step exceeds the inhibition threshold, the neuron is inhibited (i.e., $Fr_i = 0$) during that time step. The inhibition threshold is set to 15 Hz in all of our simulations; however, users can modify the inhibition threshold using a slider on the model interface.

5.4 Stochasticity

Stochasticity occurs at multiple time points during the model's initialization and procedures. During initialization, each neuron is assigned a random location within the CeA or within a specific subnucleus (CeC, CeL, or CeM) if 'Non-Uniform Distribution' is selected (Section 6).

Values of damage parameters t_S and t_L are randomly determined for each neuron using a uniform probability distribution with ranges displayed in **Table A1**. Neural connectivity is achieved by creating a network of directed links between neurons using a stochastic algorithm (Section 7.1). During each time step, a neuron's firing rate is stochastically updated using a weighted sum of values selected from truncated normal distributions (Section 7.2). Due to the stochastic nature of the model, each model simulation in our study is repeated 100 times and the mean, standard deviation, and confidence interval for all emergent output is reported.

6 Initialization

The first step in the model's initialization is the creation of CeA spatial domain. The CeA has three subnuclei: CeC, CeL, and CeM. Premade files containing the 3-dimensional coordinates of patches associated with each subnucleus are read into the model. Patches within each subnucleus are labeled and assigned the same color (CeC = red, CeL = blue, CeM = green). Next, 13,000 neurons with the variables in **Table A1** are created and assigned a location within the CeA according to either a uniform or non-uniform distribution. If the user selects 'Uniform Distribution' on the model's interface, each neuron will be randomly assigned a location within the CeA. If the user selects 'Non-Uniform Distribution' on the model's interface, then they must also specify the percentage of PKC and SST neurons within each subnucleus of the CeA using sliders on the interface. The appropriate number of PKC and SST neurons will then be randomly assigned to a location each subnucleus. Once all neurons are created and assigned a location, a network of directed links is created between the neurons.

7 Submodels

7.1 Creation of Network

The following procedures are applied to each neuron, one at a time, to create a network of uni-directional links between neurons. The selected neuron is identified as the transmitting neuron. The transmitting neuron's number of outgoing links (*Num-Out-Link)* is set to zero. An empty list is created to track the agent IDs of all neurons receiving a link from the transmitting neuron. While the transmitting neuron's *Num-Out-Link* is less than the maximum number of links allowable ($Outgoing_{max}$), the neuron will attempt to make new links with other nearby neurons. To do this, the model randomly selects a receiving neuron that is not already connected to the transmitting neuron (i.e., agent ID of receiving neuron is not the list) and is located within the maximum distance ($Dist_{max}$) of the transmitting neuron. If such a neuron exists, a directed link is created from the transmitting neuron to the receiving neuron, the connectivity variable (*Num-Out-Link*) of the transmitting neuron is incremented by 1, and the agent ID of the receiving neuron is added to the list. If no such neuron exists, no additional links are created from this transmitting neuron. The network algorithm terminates when all neurons have attained their maximum number of outgoing links or no more suitable connections can be formed.

7.2 Update of Damage Variable for Individual Neurons

A damage accumulation model is used to track a neuron's progress towards sensitization caused by noxious stimulation. During initialization, each neuron's damage level (*d*) is set equal to 0, indicating the neuron has not accumulated any damage and is unsensitized. This is the equivalent of a naïve control animal prior to injury. A neuron accrues damage only when the cumulative amount of time under stimulation exceeds the neuron's latency period ($S_{tot} > t_L$) and the current level of stimulation is greater than or equal to 120 pA. Damage stops accumulating when it reaches its maximum value ($d = 100$), indicating the neuron is sensitized. For each individual neuron, damage at time step i is updated as

$$d_i = \begin{cases} \min\left(d_{i-1} + \frac{100}{t_s}, 100\right) & \text{if } S_{tot} > t_L \text{ and } S_i \geq 120 \\ d_{i-1} & \text{if } S_{tot} \leq t_L \text{ or } S_i < 120 \end{cases} \quad (1)$$

where d_i is the value of the damage variable at time step i, t_S is the length of the neuron's sensitization period, and t_L is the length of the neuron's latency period.

7.3 Update of firing rates for individual neurons

During each time step, the firing rates of all late firing and regular spiking neurons are stochastically updated using the equation

$$Fr_i = \frac{100 - d_i}{100} \cdot X + \frac{d_i}{100} \cdot Y \quad (2)$$

where d_i is the neuron's damage level at time step i, and X and Y are random variables representing the firing rates of the neuron in an unsensitized state and a sensitized state, respectively. Both X and Y have truncated normal distributions defined by a mean, standard deviation, minimum value, and maximum value estimated from physiology data (Wilson et al. 2019, Adke et al. 2021). Parameters depend on the neuron's type (PKCδ or SST) and firing frequency (LF or RS) as well as the stimulation value (S_i). All parameter values for X and Y distributions are summarized in **Table A3**. Equation (2) is linear combination of X and Y such that when the neuron has no damage ($d = 0$), the firing rate of the unsensitized neuron is updated using the X variable only. When damage reaches its maximum value ($d = 100$), the firing rate of the sensitized neuron is updated using the Y variable only.

Spontaneous neurons fire at a constant rate of 2.838 Hz (PKCδ) and 4.887 Hz (SST) throughout each simulation (Adke et al. 2021).

7.4 Application of Network to Inhibit Neurons

After the firing rates of all PKCδ and SST neurons are updated, the neural network is used to transmit inhibitory signals between neurons in the ABM. The strength of an inhibitory signal

transmitted through a directed link is equal the firing rate of the neuron on the transmitting end. All PKCδ and SST neurons are evaluated one at a time and in a random order. For each neuron, if the total strength of all incoming signals is greater than or equal to 15 Hz, the neuron is inhibited (i.e., firing rate set to zero). If the total strength is less than 15 Hz, the neuron's firing rate does not change.

7.5 Calculation of Nociceptive Output

At the end of each time step, a system-level measure of nociceptive output (P_i) is calculated as

$$P_i = \sum_{\substack{type = PKC \\ freq = LF \text{ } or \text{ } RS}} \frac{d_i}{100} \cdot Fr_i - \sum_{\substack{type = SST \\ freq = LF \text{ } or \text{ } RS}} Fr_i \qquad (3)$$

where d_i is a neuron's damage and Fr_i is a neuron's firing rate during time step i. The first summation in equation (3) represents the weighted sum of firing rates over all PKCδ neurons that are either LF or RS. Each firing rate is weighted by the damage level of the corresponding neuron. As such, PKCδ neurons do not contribute to nociceptive output when damage is zero (i.e. pre-injury), but gradually contribute to output as sensitization occurs. When all PKCδ neurons have become sensitized (i.e., $d_i = 100$), each LF and RS PKCδ neuron contributes its firing rate to the model's output. The second summation in equation (3) represents the sum of firing rates over all SST neurons that are either late firing or regular spiking. It is assumed that SST neurons contribute to nociception at all time steps regardless of damage. Due to their anti-nociceptive properties, SST neurons are assumed to have a negative impact on the model's output.

8. Implementation

The model was coded in NetLogo3D Version 6.2.0 (Wilensky 1999). This software has a unique programming language and customizable interface that is designed specifically for ABM

development and implementation. We designed a GUI for our ABM that allows a user to easily modify parameters values, network settings, and the stimulation history. The Netlogo3D code and input files for simulating the ABM can be found in an Open Science Framework public repository (https://osf.io/xnrqa/, doi 10.17605/OSF.IO/XNRQA). For the results presented in this manuscript, we used BehaviorSpace within NetLogo3D to automate batches of 100 replicate simulations for each scenario. All graphical and statistical analyses of model output were conducted in R (R Core Team 2016).

Appendix A Tables

Table A1: Variables assigned to neuron in the ABM.

Variable	Description	Value	Frequency of updates
Loc	3-dimensial location within CeA	(x, y, z)	Assigned at initialization
Type	Protein expression type	PKCδ or SST	Assigned at initialization
Freq	Firing frequency	Late Firing (LF), Regular Spiking (RS), or Spontaneous (Spont)	Updated each time step
t_L	Length of damage latency period	integer in [40,80]	Assigned at initialization
t_S	Length of sensitizing period	integer in [50,150]	Assigned at initialization
d	Damage (percent of total damage)	real number in [0,100]	Updated each time step
Num-In-Link	Number of inputs	Integer	Assigned at initialization
Num-Out-Link	Number of outputs	Integer	Assigned at initialization
Inhibited	Inhibition status	Yes or No	Assigned at initialization and updated each time step
Fr	Firing Rate	non-negative real number	Updated each time step

Table A2: Variables assigned to each connection between neurons.

Variable	Description	Value	Frequency of updates
end1	ID of agent that is sending the inhibitory signal	positive integer	Assigned at initialization
end2	ID of agent that is receiving the inhibitory signal	positive integer	Assigned at initialization
Strength	Strength of inhibitory signal transmitted	non-negative real number	Updated each time step

Table A3: Parameters defining the probability distributions for random variables X (unsensitized firing rate) and Y (sensitized firing rate) in equation (2). Both X and Y have truncated normal distributions with mean μ, standard deviation σ, minimum value min, and maximum value max.

Type	Firing Freq.	Stimulation (pA)	Random Variable X				Random Variable Y			
			μ	σ	min	max	μ	σ	min	max
PKCδ	LF	0 – 60	0	0	0	0	0	0	0	0
PKCδ	LF	80	0	0	0	0	0.0833	0.2887	0	1
PKCδ	LF	100	0	0	0	0	0.25	0.6216	0	2
PKCδ	LF	120	0	0	0	0	0.9167	1.505	0	5
PKCδ	LF	140	0.1111	0.3234	0	1	2	2.0889	0	7
PKCδ	LF	160	0.3889	0.6978	0	2	3	2.5226	0	8
PKCδ	LF	180	0.8889	1.1827	0	3	4.25	2.958	1	10
PKCδ	LF	200	1.3889	1.4608	0	4	5.3333	2.9336	2	11
PKCδ	LF	220	2.1667	1.9778	0	6	6.75	3.1659	3	13
PKCδ	RS	0	0	0	0	0	0	0	0	0
PKCδ	RS	20	0.1111	0.4646	0	2	0.2778	0.6691	0	2
PKCδ	RS	40	0.5278	1.483	0	6	0.8333	1.6891	0	5
PKCδ	RS	60	1.2778	2.2375	0	8	1.8889	2.8052	0	7
PKCδ	RS	80	2.4444	3.1932	0	10	3.3333	3.9705	0	12
PKCδ	RS	100	3.9167	4.0169	0	13	5.3889	4.6291	0	15
PKCδ	RS	120	5.5833	4.5127	0	14	7.6111	4.6544	1	17
PKCδ	RS	140	7.1389	4.9289	0	15	9.6111	4.6417	2	19
PKCδ	RS	160	8.75	5.1513	1	16	10.571	4.6417	2	19
PKCδ	RS	180	10.0833	5.261	1	18	12.18	4.6417	2	19
PKCδ	RS	200	11.4444	5.2777	2	20	13.789	4.6417	2	19
PKCδ	RS	220	12.6389	5.3353	3	21	15.398	4.6417	2	19
SST	LF	0 - 80	0	0	0	0	0	0	0	0
SST	LF	100	0.375	1.0607	0	3	0	0	0	0
SST	LF	120	1	2.4495	0	7	0.1429	0.378	0	1
SST	LF	140	1.5	3.2071	0	9	0.4286	1.1339	0	3
SST	LF	160	2.5	4	0	11	1.1429	2.2678	0	6
SST	LF	180	3.25	4.7734	0	13	1.8571	2.9114	0	8
SST	LF	200	4.5	5.6315	0	15	3.1429	3.9761	0	11
SST	LF	220	5.625	5.7554	0	16	4.4286	4.237	0	12
SST	RS	0 – 20	0	0	0	0	0	0	0	0
SST	RS	40	0.3077	1.1094	0	4	0.45	1.2763	0	5
SST	RS	60	2.0769	2.6914	0	8	1.75	3.3067	0	11
SST	RS	80	4.6154	4.0935	0	13	3.45	4.8284	0	15
SST	RS	100	8.0769	4.9068	1	17	5.15	6.1239	0	19
SST	RS	120	11.2308	5.5551	2	22	7	7.0038	0	21
SST	RS	140	13	4.6726	4	23	8.51	7.0038	0	21
SST	RS	160	14.3077	4.9897	5	28	10.16	7.0038	0	21
SST	RS	180	15.9231	6.1028	6	30	11.81	7.0038	0	21
SST	RS	200	16.7692	6.2471	7	30	13.46	7.0038	0	21
SST	RS	220	17.1538	5.97	8	31	15.11	7.0038	0	21
