# Supplementary material for: Developing a 3-D computational model of neurons in the central amygdala to understand pharmacological targets for pain
Source: Front Pain Res (Lausanne). 2023 May 30;4:1183553. doi: 10.3389/fpain.2023.1183553 (PMC10270735; doi:10.3389/fpain.2023.1183553)
Supplement: Supplementary file 2 [file Datasheet2.pdf]

Supplemental Figures and Tables

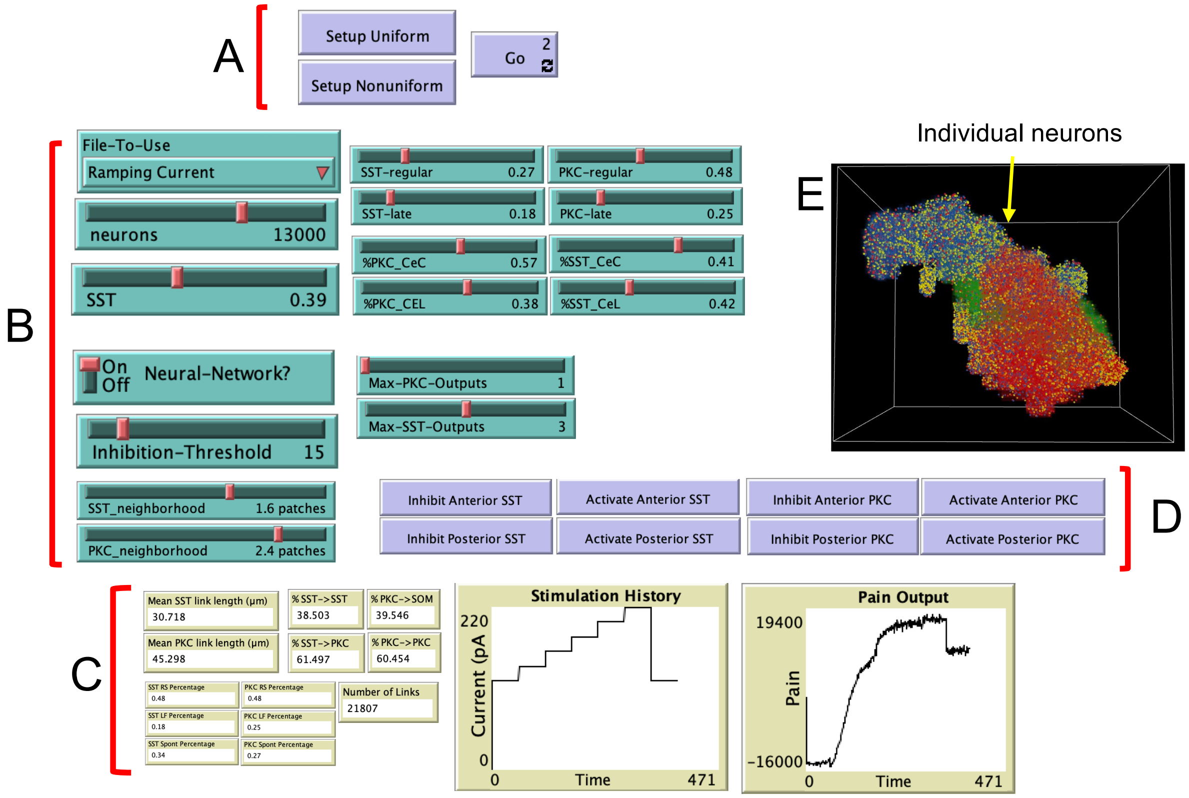

Figure S1: NetLogo3D graphical user interface for 3-D model. Panel illustrates range of buttons, switches, and sliders on NetLogo3D user interface and model output at the end of a simulation. (**A**) User can press buttons to initialize the model with either a uniform or non-uniform distribution of neurons. 'Go' button starts a model simulation. **(B)** Drop-down menu allows a user to select a stimulation file (e.g. 'Ramping Current'). User can adjust parameter values controlling the number of neurons, percentage of neurons labeled SST (all other neurons are labeled PKCδ), percentage of neurons in each subnuclei, and parameters related to the neural network. Neural network can be turned 'on' or 'off' using a toggle switch. **(C)** 3-D spatial domain of right CeA with colored patches representing the CeC (red patches), CeM (green patches), and CeL (blue patches). Individual neurons (small circles) are assigned a color based on cell-type. SST neurons are red and PKCδ neurons are blue. Neurons turn yellow if they are currently inhibited. (**D**) During a model simulation, the current (pA) and total nociceptive output ("pain") is graphed in real-time. Representative graphs shows ramping current and pain output at the end of a simulation. Panel also shows emergent network properties such as total number of links, average link length, and connectivity rates (e.g. percentage of links from SST to SST). (**E**) Buttons can be pressed at any time during a simulation to inhibit or activate select neurons in the anterior or posterior CeA.

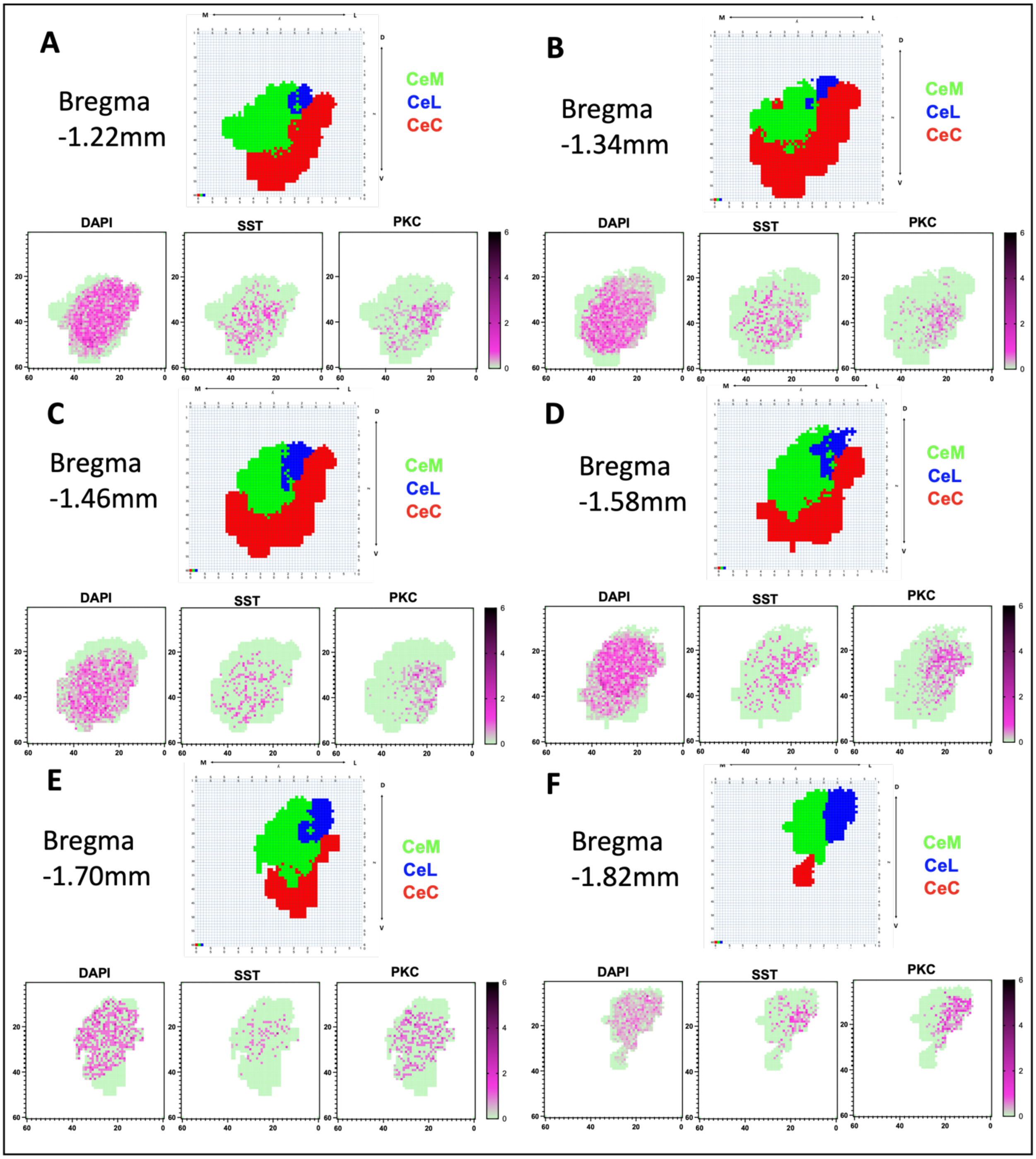

Figure S2: Quantified expression of PKC and SST in CeA. (**A-F**) PKCδ, SST, and DAPI (nuclei) were quantified from RNAScope *in situ* hybridization images across six locations anterior to posterior in the right amygdala of control wildtype mice. A heat map of the averaged data across 2-5 sections per location for DAPI, PKC-δ, and SST is shown for each indicated Bregma location within the CeA. The DAPI, PKCδ and SST positive cells that were previously marked in Figure 1A-B processing were re-counted only within an ROI established by the 2-D model slices estimated from the Blue Brain Cell Atlas. These ROI reference images of the CeA are shown for

each Bregma location. Quantified data are represented as the average number of positive cells in 25x25mm patches counted across n=5 mice.

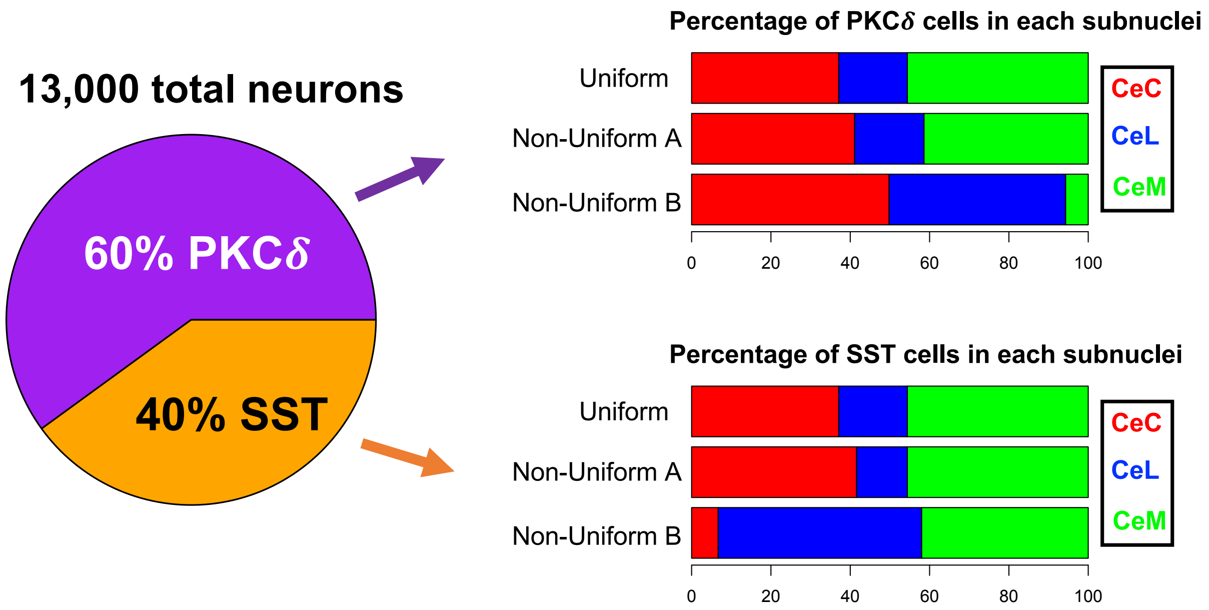

Figure S3: Overview of cell quantities and spatial distributions used in model initializations. All model simulations used 13,000 total neurons with 60% labeled PKCδ and the remaining 40% labeled SST. The only difference across the three different distributions was the localization of neurons within the respective subnuclei. In the Uniform simulations, neurons were uniformly distributed across the CeA, resulting in distributions of PKCδ and SST neurons that are proportional the relative volume of each subnuclei. In the Non-Uniform A simulations, the percentage of PKCδ and SST neurons in each subnuclei was estimated from our lab data (Section 3.1). Surprisingly, the distributions in Non-Uniform A are similar to Uniform. In the Non-Uniform B simulations, the percentage of PKCδ and SST neurons in each subnuclei was estimated published data in McCullough et al 2018. Non-Uniform B has a higher percentage of PKC neurons in the CeC/CeL and higher percentage of SST neurons within CeL/CeM than the other distributions.

Table S1: Parameter terminology used in the model in comparison to underlying wet-lab / biological phenomena.

Parameter and/or Variable Used in Model Language	Interpretation of Parameter/Variable
Select Pain-Related Output from the CeA	Difference in cumulative excitability of the PKCδ and SST neurons in the CeA
Damage/Injury/Sensitization	Change in the firing of select CeA neurons following injury or afferent stimulation
Neuronal Links	Axonal and dendritic connections between neurons
Firing Rate	Action potential firing rates from neurons derived from slice physiology experiments measured in response to current input

Table S2: Emergent network properties obtained from simulation of model for three different spatial distributions of PKCδ and SST neurons. Values represent averages (standard deviation) over 100 replicate simulations.

**Spatial distribution of neurons**	**Average total number of links** (Standard deviation)	**Average number of outgoing links per neuron**	**Average number of incoming links per neuron**
**Uniform**	22046.5<br>(42.8)	0.999 (PKCδ)<br>2.73 (SST)	1.69 (PKCδ)<br>1.69 (SST)
**Non-Uniform A**	22079.7<br>(41.4)	0.999 (PKCδ)<br>2.74 (SST)	1.70 (PKCδ)<br>1.70 (SST)
**Non-Uniform B**	20920.1*<br>(55.5)	0.999 (PKCδ)<br>2.52 (SST)*	1.51 (PKCδ)*<br>1.75 (SST)*

*Significant change in value compared to corresponding values for Uniform and Non-Uniform A ($P < 0.001$)

Table S3: Quantity of PKCδ and SST cells across A→P cross-sections. For each cross-sectional slice, quantities represent the average number of PKCδ and SST cells in each of the three subnuclei. Averages calculated from 2-5 animals per cross-sectional slice.

	**Quantity of PKCδ**			**Quantity of SST**		
**A→P cross-section**	**CeC**	**CeM**	**CeL**	**CeC**	**CeM**	**CeL**
Slice 1 (Bregma -1.22)	76.6	31	1	60	54.6	2.3
Slice 2 (Bregma -1.34)	72.7	25	2.5	66	39.5	0.75
Slice 3 (Bregma -1.46)	93	32.5	14.5	63	36.5	4.5
Slice 4 (Bregma -1.58)	55	53.6	14.3	31.3	32.6	4.3
Slice 5 (Bregma -1.70)	57	163.5	36	10.5	58	15
Slice 6 (Bregma -1.82)	2	53	83.5	3.5	36	45
